# 5-Amino-2,4,6-triiodo­isophthalic acid monohydrate

**DOI:** 10.1107/S1600536808017741

**Published:** 2008-06-19

**Authors:** Tobias Beck, George M. Sheldrick

**Affiliations:** a Department of Structural Chemistry, Georg-August Universität, Tammannstrasse 4, 37077 Göttingen, Germany

## Abstract

The title compound, C_8_H_4_I_3_NO_4_·H_2_O, shows an extensive hydrogen-bond network; in the crystal structure, mol­ecules are linked by O—H⋯O, N—H⋯O and O—H⋯N hydrogen bonds involving all possible donors and also the water mol­ecule.

## Related literature

For the synthetic procedure, see Larsen *et al.* (1956 ▶). For related crystal structure determinations: 1,3,5-triiodo­benzene, see: Margraf & Bats (2006 ▶); sodium diatrizoate, see: Tonnessen *et al.* (1996 ▶). For the 1,3,5-triiodo­benzene core as the basis of contrast agents, see: Yu & Watson (1999 ▶).
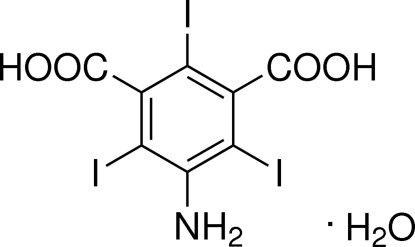


## Experimental

### 

#### Crystal data

C_8_H_4_I_3_NO_4_·H_2_O
*M* *_r_* = 576.84
Orthorhombic, 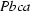

*a* = 9.214 (1) Å
*b* = 15.735 (2) Å
*c* = 18.816 (2) Å
*V* = 2728.0 (5) Å^3^
*Z* = 8
Cu *K*α radiation
μ = 54.11 mm^−1^
*T* = 100 (2) K
0.08 × 0.05 × 0.03 mm

#### Data collection

Bruker SMART 6000 diffractometer
Absorption correction: multi-scan (*SADABS*; Sheldrick, 1996 ▶) *T*_min_ = 0.106, *T*_max_ = 0.345 (expected range = 0.061–0.197)
49139 measured reflections
2716 independent reflections
2545 reflections with *I* > 2σ(*I*)
*R*_int_ = 0.043

#### Refinement

*R*[*F* ^2^ > 2σ(*F* ^2^)] = 0.026
*wR*(*F* ^2^) = 0.061
*S* = 1.03
2716 reflections
173 parameters
14 restraints
Only H-atom coordinates refined
Δρ_max_ = 0.71 e Å^−3^
Δρ_min_ = −1.71 e Å^−3^

### 

Data collection: *APEX2* (Bruker, 2007 ▶); cell refinement: *SAINT* (Bruker, 2007 ▶); data reduction: *SAINT*; program(s) used to solve structure: *SHELXS97* (Sheldrick, 2008 ▶); program(s) used to refine structure: *SHELXL97* (Sheldrick, 2008 ▶); molecular graphics: *SHELXTL* (Sheldrick, 2008 ▶); software used to prepare material for publication: *SHELXL97* and *SHELXTL*.

## Supplementary Material

Crystal structure: contains datablocks global, I. DOI: 10.1107/S1600536808017741/pk2102sup1.cif

Structure factors: contains datablocks I. DOI: 10.1107/S1600536808017741/pk2102Isup2.hkl

Additional supplementary materials:  crystallographic information; 3D view; checkCIF report

## Figures and Tables

**Table 1:** Hydrogen-bond geometry (Å, °)

*D*—H⋯*A*	*D*—H	H⋯*A*	*D*⋯*A*	*D*—H⋯*A*
O11—H11⋯O14	0.79 (5)	1.75 (5)	2.540 (5)	173 (7)
O8—H8⋯O12^i^	0.80 (5)	1.90 (5)	2.662 (5)	161 (7)
O14—H14*A*⋯O9^ii^	0.81 (4)	1.95 (4)	2.751 (5)	170 (6)
O14—H14*B*⋯N13^iii^	0.81 (4)	2.05 (4)	2.841 (5)	166 (6)
N13—H13*A*⋯O14^iv^	0.88 (4)	2.30 (5)	3.067 (6)	147 (5)
N13—H13*B*⋯O12^iv^	0.88 (4)	2.68 (5)	3.478 (5)	152 (5)

Symmetry codes: (i) 
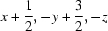
; (ii) 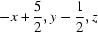; (iii) 
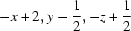
; (iv) 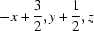.

**Fig. 1.**
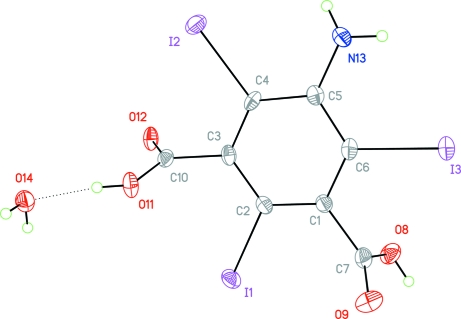
A view of I3C. Displacement ellipsoids are drawn at the 50% probability level. The hydrogen bond within the asymmetric unit is shown as a dashed line.

**Fig. 2.**
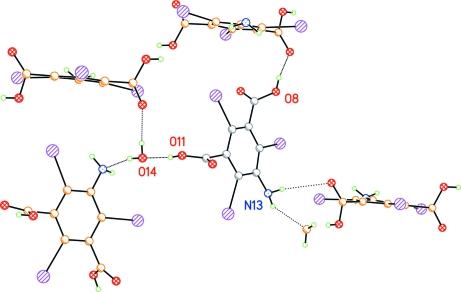
Hydrogen bonding of I3C. Symmetry equivalents are depicted in orange.

**Fig. 3.**
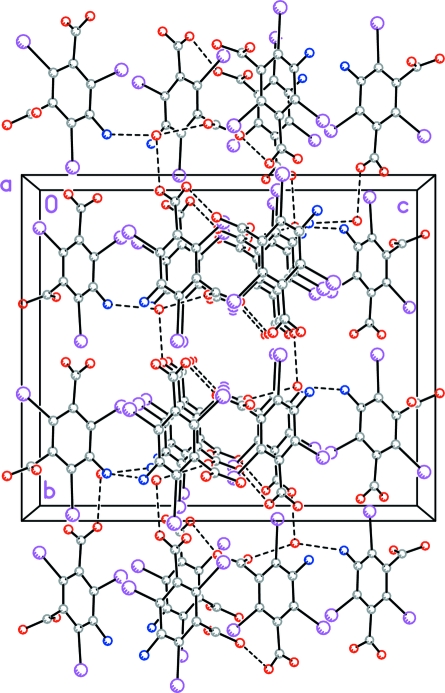
Packing of I3C, viewed along b. Hydrogen atoms are omitted for clarity. In alternating layers molecules are positioned perpendicular to each other. Hydrogen bonds are shown as dashed lines.

**Fig. 4.**
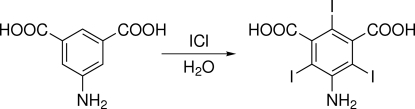
Synthetic scheme of I3C.

## supplementary crystallographic information

### Comment

Iodine-based compounds have always been in the focus of contrast agents for X-ray imaging. The 1,3,5-triiodobenzene core has been the basis of many contrast agents (Yu & Watson 1999). The ionic monomer diatrizoate was one of the first compounds used (Tonnessen *et al.* 1996).

The title compound, 5-Amino-2,4,6-triiodoisophthalic acid (hereafter I3C), crystallizes as a monohydrate, due to water impurities in the crystallization solution. It forms hydrogen bonds with all potential donors as well as the lattice water being involved (Fig. 2, Table 1). However, the interaction between N13 and O12 is slightly weaker. In the crystal, the molecules are positioned perpendicular to each other, showing no π-π interactions of the phenyl rings (Fig. 3).

The three functional groups for hydrogen bonding, along with the three iodine atoms, render I3C a suitable agent for experimental phasing of macromolecules (Beck *et al.*, unpublished results). The iodine atoms give rise to a large anomalous signal, even at in-house sources. Additionally, they form an equilateral triangle (I—I 6.0 Å) which is easy to recognize in the heavy atom substructure when this compound is used as a heavy atom derivative for macromolecular phasing.

### Experimental

The title compound was prepared according to the reported procedure (Larsen *et al.* 1956). It was recrystallized from a methanol-acetonitrile solution by slowly evaporating the solvents to obtain crystals suitable for X-ray single-crystal diffraction.

### Refinement

Hydrogen atoms were located *via* the difference Fourier map and their geometrical positions were refined with restraints. The *U* values were set to 1.5 *U*_eq_ of their parent atom. Bond lengths for hydrogen atoms were restrained to be equal (SADI in SHELXL-97). Phenyl ring and carboxylate groups were restrained to planarity.

### Crystal data

**Table:**

C_8_H_4_I_3_NO_4_·H_2_O	*F*_000_ = 2080
*M**_r_* = 576.84	*D*_x_ = 2.809 Mg m^−^^3^
Orthorhombic, *P**b**c**a*	Cu *K*α radiation λ = 1.54178 Å
Hall symbol: -P 2ac 2ab	Cell parameters from 9945 reflections
*a* = 9.214 (1) Å	θ = 4.7–60.8º
*b* = 15.735 (2) Å	µ = 54.11 mm^−^^1^
*c* = 18.816 (2) Å	*T* = 100 (2) K
*V* = 2728.0 (5) Å^3^	Block, yellow
*Z* = 8	0.08 × 0.05 × 0.03 mm

### Data collection

**Table:**

Bruker SMART 6000 diffractometer	2716 independent reflections
Radiation source: rotating anode	2545 reflections with *I* > 2σ(*I*)
Monochromator: INCOATEC multilayer optics	*R*_int_ = 0.043
Detector resolution: 5.602 pixels mm^-1^	θ_max_ = 74.3º
*T* = 100(2) K	θ_min_ = 4.7º
ω scans	*h* = −10→10
Absorption correction: multi-scan(SADABS; Sheldrick, 1996)	*k* = −17→19
*T*_min_ = 0.106, *T*_max_ = 0.345	*l* = −23→23
49139 measured reflections	

### Refinement

**Table:**

Refinement on *F*^2^	Secondary atom site location: difference Fourier map
Least-squares matrix: full	Hydrogen site location: difference Fourier map
*R*[*F*^2^ > 2σ(*F*^2^)] = 0.026	Only H-atom coordinates refined
*wR*(*F*^2^) = 0.061	*w* = 1/[σ^2^(*F*_o_^2^) + (0.0277*P*)^2^ + 17.3624*P*] where *P* = (*F*_o_^2^ + 2*F*_c_^2^)/3
*S* = 1.03	(Δ/σ)_max_ = 0.002
2716 reflections	Δρ_max_ = 0.72 e Å^−^^3^
173 parameters	Δρ_min_ = −1.71 e Å^−^^3^
14 restraints	Extinction correction: SHELXL97 (Sheldrick, 2008), Fc^*^=kFc[1+0.001xFc^2^λ^3^/sin(2θ)]^-1/4^
Primary atom site location: structure-invariant direct methods	Extinction coefficient: 0.000058 (8)

### Special details

**Table:**

Experimental. Intensities were measured with a Bruker *SMART* 6000 area-detector
Geometry. All e.s.d.'s (except the e.s.d. in the dihedral angle between two l.s. planes) are estimated using the full covariance matrix. The cell e.s.d.'s are taken into account individually in the estimation of e.s.d.'s in distances, angles and torsion angles; correlations between e.s.d.'s in cell parameters are only used when they are defined by crystal symmetry. An approximate (isotropic) treatment of cell e.s.d.'s is used for estimating e.s.d.'s involving l.s. planes.
Refinement. Refinement of *F*^2^ against ALL reflections. The weighted *R*-factor *wR* and goodness of fit *S* are based on *F*^2^, conventional *R*-factors *R* are based on *F*, with *F* set to zero for negative *F*^2^. The threshold expression of *F*^2^ > 2 σ(*F*^2^) is used only for calculating *R*-factors(gt) *etc*. and is not relevant to the choice of reflections for refinement. *R*-factors based on *F*^2^ are statistically about twice as large as those based on *F*, and *R*- factors based on ALL data will be even larger. Hydrogen atoms were located *via* the difference Fourier map and their geometrical positions were refined with restraints. The *U* values were set to 1.5 *U*_eq_ of their parent atom.

### Fractional atomic coordinates and isotropic or equivalent isotropic displacement parameters (Å^2^)

**Table:**

	*x*	*y*	*z*	*U*_iso_*/*U*_eq_	
I1	1.18424 (3)	0.630616 (18)	0.012748 (16)	0.02419 (10)	
I2	0.71499 (3)	0.674286 (18)	0.234093 (15)	0.02375 (10)	
I3	1.04238 (3)	0.979824 (17)	0.124476 (17)	0.02693 (10)	
C1	1.0827 (5)	0.7970 (3)	0.0781 (2)	0.0161 (8)	
C2	1.0606 (5)	0.7094 (3)	0.0789 (2)	0.0161 (9)	
C3	0.9544 (5)	0.6745 (3)	0.1231 (2)	0.0153 (9)	
C4	0.8655 (5)	0.7276 (3)	0.1631 (2)	0.0163 (9)	
C5	0.8803 (5)	0.8172 (3)	0.1604 (2)	0.0165 (9)	
C6	0.9945 (5)	0.8492 (3)	0.1199 (2)	0.0180 (9)	
C7	1.1981 (5)	0.8362 (3)	0.0320 (3)	0.0202 (10)	
O8	1.1458 (4)	0.8648 (2)	−0.02803 (18)	0.0256 (7)	
H8	1.205 (6)	0.883 (4)	−0.055 (3)	0.038*	
O9	1.3242 (4)	0.8414 (2)	0.05017 (19)	0.0290 (8)	
C10	0.9364 (5)	0.5794 (3)	0.1285 (2)	0.0173 (9)	
O11	1.0376 (4)	0.5445 (2)	0.16663 (17)	0.0224 (7)	
H11	1.031 (7)	0.494 (3)	0.170 (3)	0.034*	
O12	0.8358 (4)	0.54161 (19)	0.10032 (17)	0.0213 (7)	
N13	0.7889 (5)	0.8702 (2)	0.1997 (2)	0.0210 (8)	
H13A	0.698 (5)	0.855 (4)	0.204 (3)	0.031*	
H13B	0.785 (6)	0.922 (3)	0.183 (3)	0.031*	
O14	1.0395 (4)	0.3834 (2)	0.17540 (18)	0.0235 (7)	
H14A	1.074 (7)	0.366 (4)	0.139 (3)	0.035*	
H14B	1.099 (6)	0.376 (4)	0.206 (3)	0.035*	

### Atomic displacement parameters (Å^2^)

**Table:**

	*U*^11^	*U*^22^	*U*^33^	*U*^12^	*U*^13^	*U*^23^
I1	0.02850 (18)	0.02100 (16)	0.02307 (16)	0.00785 (12)	0.00724 (12)	0.00027 (11)
I2	0.02603 (18)	0.02156 (16)	0.02364 (16)	−0.00544 (11)	0.00910 (12)	−0.00016 (11)
I3	0.02852 (19)	0.01260 (15)	0.03966 (19)	−0.00146 (11)	0.00662 (13)	0.00096 (11)
C1	0.018 (2)	0.0143 (19)	0.0162 (19)	0.0012 (17)	0.0007 (17)	0.0000 (16)
C2	0.019 (2)	0.014 (2)	0.0150 (19)	0.0033 (17)	−0.0002 (17)	−0.0002 (16)
C3	0.020 (2)	0.013 (2)	0.0135 (19)	0.0006 (17)	−0.0052 (17)	0.0019 (15)
C4	0.018 (2)	0.016 (2)	0.0150 (19)	−0.0072 (16)	0.0005 (17)	0.0017 (16)
C5	0.018 (2)	0.015 (2)	0.016 (2)	0.0003 (17)	−0.0023 (17)	−0.0006 (16)
C6	0.023 (2)	0.013 (2)	0.017 (2)	−0.0022 (18)	−0.0013 (18)	0.0009 (16)
C7	0.023 (3)	0.014 (2)	0.023 (2)	−0.0007 (17)	0.0010 (19)	−0.0016 (17)
O8	0.0251 (19)	0.0276 (18)	0.0241 (17)	−0.0019 (14)	0.0036 (15)	0.0093 (14)
O9	0.0216 (19)	0.038 (2)	0.0276 (18)	−0.0045 (15)	0.0035 (15)	0.0013 (15)
C10	0.023 (2)	0.014 (2)	0.015 (2)	−0.0007 (18)	0.0048 (18)	0.0001 (16)
O11	0.0274 (18)	0.0132 (15)	0.0265 (17)	−0.0007 (13)	−0.0074 (14)	0.0043 (13)
O12	0.0279 (18)	0.0121 (14)	0.0239 (16)	−0.0039 (13)	−0.0064 (14)	0.0014 (12)
N13	0.021 (2)	0.0148 (18)	0.027 (2)	0.0019 (15)	0.0035 (17)	−0.0026 (15)
O14	0.0255 (19)	0.0217 (16)	0.0233 (17)	0.0028 (14)	−0.0003 (14)	0.0027 (14)

### Geometric parameters (Å, °)

**Table:**

I1—C2	2.094 (4)	C5—N13	1.396 (6)
I2—C4	2.100 (4)	C7—O9	1.214 (6)
I3—C6	2.103 (4)	C7—O8	1.308 (6)
C1—C2	1.394 (6)	O8—H8	0.80 (5)
C1—C6	1.398 (6)	C10—O12	1.223 (6)
C1—C7	1.504 (6)	C10—O11	1.299 (6)
C2—C3	1.396 (6)	O11—H11	0.79 (5)
C3—C4	1.391 (6)	N13—H13A	0.88 (4)
C3—C10	1.509 (6)	N13—H13B	0.88 (4)
C4—C5	1.418 (6)	O14—H14A	0.81 (4)
C5—C6	1.393 (6)	O14—H14B	0.81 (4)
			
C2—C1—C6	119.4 (4)	C5—C6—C1	122.3 (4)
C2—C1—C7	121.0 (4)	C5—C6—I3	119.3 (3)
C6—C1—C7	119.6 (4)	C1—C6—I3	118.4 (3)
C1—C2—C3	119.8 (4)	O9—C7—O8	125.0 (4)
C1—C2—I1	120.0 (3)	O9—C7—C1	122.7 (4)
C3—C2—I1	120.2 (3)	O8—C7—C1	112.3 (4)
C4—C3—C2	120.0 (4)	C7—O8—H8	115 (5)
C4—C3—C10	119.6 (4)	O12—C10—O11	125.3 (4)
C2—C3—C10	120.4 (4)	O12—C10—C3	122.5 (4)
C3—C4—C5	121.3 (4)	O11—C10—C3	112.3 (4)
C3—C4—I2	119.6 (3)	C10—O11—H11	114 (5)
C5—C4—I2	119.0 (3)	C5—N13—H13A	117 (4)
C6—C5—N13	122.0 (4)	C5—N13—H13B	113 (4)
C6—C5—C4	116.9 (4)	H13A—N13—H13B	105 (6)
N13—C5—C4	121.0 (4)	H14A—O14—H14B	108 (6)
			
C6—C1—C2—C3	−2.2 (6)	N13—C5—C6—C1	−177.4 (4)
C7—C1—C2—C3	179.0 (4)	C4—C5—C6—C1	6.0 (6)
C6—C1—C2—I1	176.7 (3)	N13—C5—C6—I3	5.2 (5)
C7—C1—C2—I1	−2.0 (5)	C4—C5—C6—I3	−171.4 (3)
C1—C2—C3—C4	3.5 (6)	C2—C1—C6—C5	−2.8 (6)
I1—C2—C3—C4	−175.4 (3)	C7—C1—C6—C5	176.0 (4)
C1—C2—C3—C10	−175.6 (4)	C2—C1—C6—I3	174.7 (3)
I1—C2—C3—C10	5.5 (5)	C7—C1—C6—I3	−6.5 (5)
C2—C3—C4—C5	−0.1 (6)	C2—C1—C7—O9	−84.0 (6)
C10—C3—C4—C5	179.1 (4)	C6—C1—C7—O9	97.2 (5)
C2—C3—C4—I2	−176.2 (3)	C2—C1—C7—O8	97.3 (5)
C10—C3—C4—I2	3.0 (5)	C6—C1—C7—O8	−81.5 (5)
C3—C4—C5—C6	−4.6 (6)	C4—C3—C10—O12	75.4 (5)
I2—C4—C5—C6	171.5 (3)	C2—C3—C10—O12	−105.5 (5)
C3—C4—C5—N13	178.8 (4)	C4—C3—C10—O11	−103.6 (4)
I2—C4—C5—N13	−5.1 (5)	C2—C3—C10—O11	75.5 (5)

### Hydrogen-bond geometry (Å, °)

**Table:**

*D*—H···*A*	*D*—H	H···*A*	*D*···*A*	*D*—H···*A*
O11—H11···O14	0.79 (5)	1.75 (5)	2.540 (5)	173 (7)
O8—H8···O12^i^	0.80 (5)	1.90 (5)	2.662 (5)	161 (7)
O14—H14A···O9^ii^	0.81 (4)	1.95 (4)	2.751 (5)	170 (6)
O14—H14B···N13^iii^	0.81 (4)	2.05 (4)	2.841 (5)	166 (6)
N13—H13A···O14^iv^	0.88 (4)	2.30 (5)	3.067 (6)	147 (5)
N13—H13B···O12^iv^	0.88 (4)	2.68 (5)	3.478 (5)	152 (5)

Symmetry codes: (i) *x*+1/2, −*y*+3/2, −*z*; (ii) −*x*+5/2, *y*−1/2, *z*; (iii) −*x*+2, *y*−1/2, −*z*+1/2; (iv) −*x*+3/2, *y*+1/2, *z*.
